# Understanding the Complex Dynamics of Immunosenescence in Multiple Sclerosis: From Pathogenesis to Treatment

**DOI:** 10.3390/biomedicines12081890

**Published:** 2024-08-19

**Authors:** Monica Neațu, Ana Hera-Drăguț, Iulia Ioniță, Ana Jugurt, Eugenia Irene Davidescu, Bogdan Ovidiu Popescu

**Affiliations:** 1 Department of Clinical Neurosciences, “Carol Davila” University of Medicine and Pharmacy, 050474 Bucharest, Romania; monica.neatu@rez.umfcd.ro (M.N.); ana-marina.hera@rez.umfcd.ro (A.H.-D.); iulia.ionita@rez.umfcd.ro (I.I.); ana.jugurt@rez.umfcd.ro (A.J.); bogdan.popescu@umfcd.ro (B.O.P.); 2 Department of Neurology, Colentina Clinical Hospital, 020125 Bucharest, Romania; 3 Department of Cell Biology, Neurosciences and Experimental Myology, “Victor Babeș” National Institute of Pathology, 050096 Bucharest, Romania

**Keywords:** immunosenescence, multiple sclerosis, inflammaging, aging, neuroinflammation

## Abstract

Immunosenescence, the gradual deterioration of immune function with age, holds profound implications for our understanding and management of multiple sclerosis (MS), a chronic autoimmune disease affecting the central nervous system. Traditionally diagnosed in young adults, advancements in disease-modifying therapies and increased life expectancy have led to a growing number of older individuals with MS. This demographic shift underscores the need for a deeper investigation into how age-related alterations in immune function shape the course of MS, influencing disease progression, treatment effectiveness, and overall patient outcomes. Age-related immunosenescence involves changes such as shifts in cytokine profiles, the accumulation of senescent immune cells, and compromised immune surveillance, collectively contributing to a state known as “inflammaging”. In the context of MS, these immunological changes disturb the intricate balance between inflammatory and regulatory responses, thereby impacting mechanisms of central immune tolerance and peripheral regulation. This paper stands out by combining the most recent advancements in immunosenescence with both pathophysiological and treatment perspectives on multiple sclerosis, offering a cohesive and accessible discussion that bridges theory and practice, while also introducing novel insights into underexplored concepts such as therapy discontinuation and the latest senolytic, neuroprotective, and remyelination therapies. Enhancing our understanding of these complexities will guide tailored approaches to MS management, ultimately improving clinical outcomes for affected individuals.

## 1. Introduction

Multiple sclerosis (MS) is a chronic, inflammatory, demyelinating autoimmune disorder that affects the central nervous system (CNS). It is well established that the immune system is implicated in the destruction of myelin sheaths, resulting in progressive neurological disability in patients. Despite substantial advancements in elucidating the pathophysiology of MS, the precise etiology in the initial stages of the disease remains incompletely understood. It predominantly affects young adults, and it is more prevalent among women. Recent advances in MS treatments and the management of age-related comorbidities have significantly increased the life expectancy of MS patients, such that it now approaches that of the general population. This has resulted in a considerable increase in the number of older patients living with MS [1,2,3,4,5,6].

The gradual decline of the immune system associated with aging involves significant changes such as thymic involution, which reduces the production of naive T cells; an inverted CD4+/CD8+ cell ratio; impaired natural killer (NK) cell function; and diminished tissue repair capacity. Additionally, aging increases the activity of immunosuppressive cells like regulatory T (Treg) cells and regulatory B (Breg) cells. Alterations in both adaptive and innate immunity will be examined in detail in the upcoming sections. These changes lead to increased morbidity and mortality in older adults by influencing various inflammatory processes, heightening their susceptibility to infections, and increasing the risk of autoimmune diseases and neurodegenerative conditions [7,8].

In the context of MS, immunosenescence may accelerate disease progression and complicate treatment, particularly as the average age of MS patients continues to rise. Understanding the underlying mechanisms of immunosenescence is critical for developing new therapies and enhancing outcomes for older individuals with MS. This paper takes a significant step forward by bringing together the latest knowledge on immunosenescence, particularly in relation to multiple sclerosis. Unlike earlier studies that tended to focus on either the pathophysiology or treatment aspects separately, our work integrates both perspectives into a single, cohesive discussion. This dual approach not only makes complex ideas more accessible to professionals, but it also helps to bridge the gap between theory and practical application. Also, by highlighting the role of therapy discontinuation and de-escalation, we shine a light on key concepts that have not been fully explored. Furthermore, our paper introduces the latest findings on senolytic, neuroprotective, and remyelination therapies, presenting a novel perspective on their relevance to multiple sclerosis [5,6,7].

## 2. Immune System Aging

The aging of the immune system, known as immunosenescence, results in diminished immune responses among older adults. These changes not only impair the functionality of immune cells but also alter their number and frequency due to reduced hematopoiesis and progressive thymic atrophy. Hematopoietic stem cells (HSCs), crucial for generating immune cells in the bone marrow, exhibit impaired telomerase activity and telomere shortening with each division, leading to a decline in their self-renewal capacity over time. Furthermore, aging HSCs show a preference for myeloid over lymphoid differentiation, which has an overall impact on the immune function [9,10].

### 2.1. Immunosenescence: Adaptive Immunity

The adaptive immune system consists of humoral immunity, which is managed by activated B cells and antibodies, and cell-mediated immunity, which is governed by T cells. Within T cells, CD4+ T cells play a crucial role in regulating immune responses against pathogens by differentiating into different T helper (Th) cell lineages (Th1, Th2, Th17, and Treg), each characterized by specific cytokine profiles and functions. Meanwhile, CD8+ T cells contribute to the clearance of intracellular pathogens and provide long-term immune protection [10,11,12,13] (Table 1).

#### 2.1.1. Immunosenescence: T Cells

T cell changes are a prominent aspect of immunosenescence. One of the most significant effects of aging is the progressive involution of the thymus, which severely impacts the maturation of naive T cells and reduces the diversity of the T cell receptor (TCR) repertoire. This thymic involution can be quantified using TRECs (T cell receptor excision circles) as biomarkers. These circular DNA fragments, produced during TCR gene rearrangement in the thymus, are found exclusively in naive T cells [11,12,13,14].

In older adults, although the overall T cell population remains stable, the number of naive T cells, particularly CD8+ T cells, decreases significantly in the periphery. Concurrently, there is an expansion and accumulation of terminally differentiated memory T cells due to persistent infections such as cytomegalovirus (CMV). This results in a reduced diversity of the TCR repertoire, impairing the T cell-mediated immune response and diminishing the ability to respond effectively to infections and vaccinations [11,12,13,14,15].

Senescent T cells are characterized by the loss of the CD28 co-stimulatory molecule and the expression of senescence markers like CD57 and KLRG1. These features are typical of terminally differentiated memory CD8+ T cells in elderly individuals. These cells exhibit a reduced capacity to proliferate, increased resistance to apoptosis, and shortened telomeres, hallmark features of cellular senescence. Additionally, senescent CD8+ T cells express inhibitory receptors such as TIGIT and PD1, which are indicative of both senescent and exhausted T cells. While these terminally differentiated memory CD8+ T cells can combat persistent infections in older adults, they also secrete a specific cytokine profile known as the senescence-associated secretory phenotype (SASP). SASP contributes to further tissue damage, as we will discuss in more detail below [12,14,15,16,17,18,19,20].

Moreover, a subset of CD8+ T cells constitutively express the NK-associated receptor NKG2D, which serves as an activating receptor in cytotoxic cells. As CD8+ T cells age and lose CD28 expression, they accumulate NK receptors, transitioning from TCR- to NK receptor-mediated cytotoxicity. This switch likely enhances their capacity for immune surveillance and the elimination of senescent and tumor cells in aged tissues. Conversely, CD4+ CD28− T cells express NK receptors de novo and expand in certain autoimmune conditions. In aged individuals, these cells, specifically those expressing the NKG2D receptor, are expanded and thus are considered differentiation markers in the CD4+ T cell compartment. This acquisition of innate-like properties by senescent T cells blurs the boundaries between innate and adaptive immunity [13,21,22,23,24].

Although the most significant changes occur in the CD8+ T cell compartment, CD4+ T cells also undergo modifications with age, though to a lesser extent. Terminally differentiated CD4+ T cells display similar senescence features, such as their accumulation due to persistent antigen stimulation, shortened telomeres, and high production of pro-inflammatory cytokines. These cells also express the senescence markers CD57 and KLRG1 and lose the CD28 co-stimulatory molecule. In senescent CD4+ T cells, there is an increase in autoreactive cells. The loss of CD28 impairs their ability to induce the expression of CD40L, which is crucial for interacting with CD40 molecules on B cells. Consequently, CD4+ T cells lose their helper function, reducing their ability to promote B cell proliferation and antibody production [12,19,20,21,22,23,24,25,26,27].

#### 2.1.2. Immunosenescence: B Cells

Although research on immunosenescence has predominantly described T cell dysfunction, B cells also experience substantial alterations (Table 1). Despite the observed shift toward myeloid differentiation in the bone marrow, as mentioned earlier, the total count of B cells in peripheral blood remains relatively stable. Nevertheless, there are significant shifts in the composition of B cell subpopulations that are characterized by reduced diversity, altered B cell receptor repertoires, and an increased proportion of memory cells, which contribute to a compromised humoral immune response in older adults [11,12,28,29,30].

Aging leads to a decrease in the production of naive B cells and a shift from naive (CD27−) to memory B cells (CD27+). Late memory B cells become more numerous and express markers associated with migration to sites of inflammation. Additionally, the isotype switch from IgM to IgG, IgE, or IgA occurs less frequently in the elderly [28].

A specific subset of B cells, known as age-associated B cells (ABCs), expands with age. ABCs are activated through Toll-like receptors (TLR) 7 and TLR9 and possess features of memory B cells. These cells tend to produce antibodies against self-antigens and are commonly encountered in individuals suffering from autoimmune conditions. ABCs adopt a pro-inflammatory phenotype, secrete more IFNγ and TNF-α (inhibiting bone marrow B cell development), and promote the differentiation of Th17 cells [12,28,30,31,32,33].

### 2.2. Immunosenescence: Innate Immunity

The innate immune cells, primarily represented by neutrophils, monocytes, natural killer cells, and dendritic cells (DCs), provide an immediate, non-antigen-specific response to potential viral, bacterial, and fungal pathogens. These molecules rely on pattern-recognition receptors (PRRs) to identify and react to microbes and damaged cells. There are three classes of PRRs (Table 2). Their role is to recognize and respond to exogenous microbial pathogen-associated molecular patterns (PAMPs) (e.g., viral RNA, lipoteichoic acid), as well as endogenous cellular debris and damage-associated molecular patterns (DAMPs) (e.g., S100 proteins, heat shock proteins) [10,34,35,36,37] (Table 3).

#### 2.2.1. Immunosenescence: Neutrophils

Neutrophils, essential components of the innate immune system, play a crucial role in the initial defense against bacterial and fungal infections. They employ various mechanisms such as the production of reactive oxygen species (ROS), secretion of antimicrobial peptides, and formation of neutrophil extracellular traps (NETosis) to combat pathogens. Although the absolute number of neutrophils in older adults does not differ from that of the younger population, significant alterations in neutrophil function occur (Table 3). For instance, aged neutrophils exhibit reduced ROS production and NET formation, which contribute to increased susceptibility to infections among the elderly. Furthermore, age-related modifications in neutrophil surface molecules such as CD11b and CD16 impair their ability to perform crucial functions like phagocytosis and adhesion to endothelial cells. This impairment limits their capacity to migrate effectively to sites and to participate in immune cell recruitment [10,11,38,39,40,41,42].

#### 2.2.2. Immunosenescence: Monocytes and Macrophages

Monocytes are circulating cells recruited to the inflammation site, where they can differentiate into macrophages. After activation, macrophages divide into two subgroups: M1-like macrophages that produce pro-inflammatory cytokines such as IL-1, IL-6 and TNF-α and are mostly associated with pro-inflammatory responses; and M2-like macrophages that secrete high levels of IL-10 and TGF-beta and promote anti-inflammatory and cell proliferation responses. Aging does not significantly alter the absolute number of monocytes in humans, but it does affect their subsets and functionality (Table 3). The aging process determines not only a functional decline (impaired migration, chemotaxis, and phagocytosis) but also morphological abnormalities (increased cell size). Moreover, there appears to be a shift toward M2 cells rather than the classic M1 type. Another age-related dysfunction of macrophages is the reduced expression of TLRs, which affects their ability to recognize pathogens and to activate appropriate inflammatory responses. This dysregulation includes diminished TLR1 expression and altered signaling pathways, leading to impaired cytokine production in response to pathogens [11,38,43,44,45,46,47].

#### 2.2.3. Immunosenescence: Natural Killer Cells

Natural killer (NK) cells are essential components of the innate immune system, playing a key role in cancer surveillance and defense against intracellular pathogens. Moreover, these cells regulate the immune response by producing cytokines such as TNF, INF, and IL-5. They are identified by their expression of CD56 and/or CD16 and can be categorized into two subsets based on these markers. The CD56^bright^CD16^neg/dim^ subset consists of immature cells and produces cytokines and chemokines, while the CD56^dim^CD16+ subset consists of mature NK cells with high cytotoxic ability. With aging, the absolute number of NK cells remains the same or tends to increase slightly, possibly as a compensatory response. However, despite this numerical stability, the function and phenotype of NK cells can be compromised, leading to reduced cytotoxic activity, cytokine production, and proliferative capacity (Table 3). Aging is also associated with defective degranulation due to a shift in NK cell subpopulations, with an increase in CD56^dim^ cells and a decrease in CD56^bright^ cells [11,38,43,48,49].

#### 2.2.4. Immunosenescence: Dendritic Cells

Dendritic cells (DCs) link the innate and adaptive immunity by presenting antigens to naive T cells, thereby influencing the differentiation of CD4+ T-cells into various phenotypes such as Th1 and Th2. Aging induces significant alterations in DCs, including mitochondrial dysfunction and increased ROS production, which impair their phagocytic capabilities and antigen presentation (Table 3). This dysfunction specifically affects the activation and cytotoxic response of CD8+ T cells and diminishes the secretion of IFN-γ [10,11,38].

One hypothesis for these age-related changes in DCs involves excessive NF-κB stimulation driven by cellular senescence. These functional alterations have been attributed to decreased PI3K activity, leading to alternative signaling events and the upregulation of NF-κB, resulting in the abnormal production of pro-inflammatory cytokines such as TNF-α and IL-6 [43,50,51].

There are two primary types of dendritic cells: conventional/myeloid dendritic cells (mDCs) and plasmacytoid dendritic cells (pDCs). mDCs produce IL-12, which is crucial for Th1 and cytotoxic T lymphocyte (CTL) responses, while pDCs produce IFN-α/β in response to bacterial or viral infections. Both types are vital for presenting antigens to naive T cells and influencing their polarization. Research indicates that the number of circulating pDCs and mDCs decreases in elderly individuals, especially women. Also, DCs show reduced responsiveness to TLR stimulation, although this is not consistent across all tissues, suggesting some variability in cell distribution with age. Monocyte-derived dendritic cells, considered by some to be a third subtype, also display reduced functionality in older individuals [10,11,38,43,50].

## 3. Immunosenescence and Inflammaging

The existence of “inflammaging”, a persistent low-grade inflammation that is correlated with higher morbidity and mortality, is a prevalent hallmark of age-related illnesses and tissue aging. Its etiopathogenesis is still far from being fully understood. To better understand inflammaging and how all the cellular and molecular alterations described above contribute to this phenomenon, we highlight the following [11,52,53].

Age-related senescent cells tend to aggregate into end-stage differentiated senescent T cells, defined by proliferative arrest, and differentiated CD28 T cells, produced by a repetitive pathogen encounter. Although it was once thought that these cells were dormant, recent data have demonstrated that they are metabolically active. The senescence-associated secretory phenotype, or SASP, is a condition in which the body produces high amounts of pro-inflammatory cytokines (Table 4) [43,54,55,56,57,58].

This persistent antigenic stimulation, which causes a rise in senescent T cells and impairs CD4+ cells, leads to numerous dysregulated responses that promote inflammaging. Two other mechanisms leading to inflammaging include the increased ratio of pro-inflammatory Th17 cells to immunosuppressive T regulatory cells, which favors a pro-inflammatory state; and the decreased assistance to B cells, which results in decreased humoral immunity. Therefore, the imbalance between the pro-inflammatory and anti-inflammatory immune responses are caused by changes in the TH17/Treg ratios and altered cytokine expression, suggesting a higher risk of acquiring inflammatory disorders. According to reports, unstimulated human B lymphocytes from aged adults can release more TNF-α, a characteristic of inflammaging [43,54,55,56,57,58].

**Figure 1:**
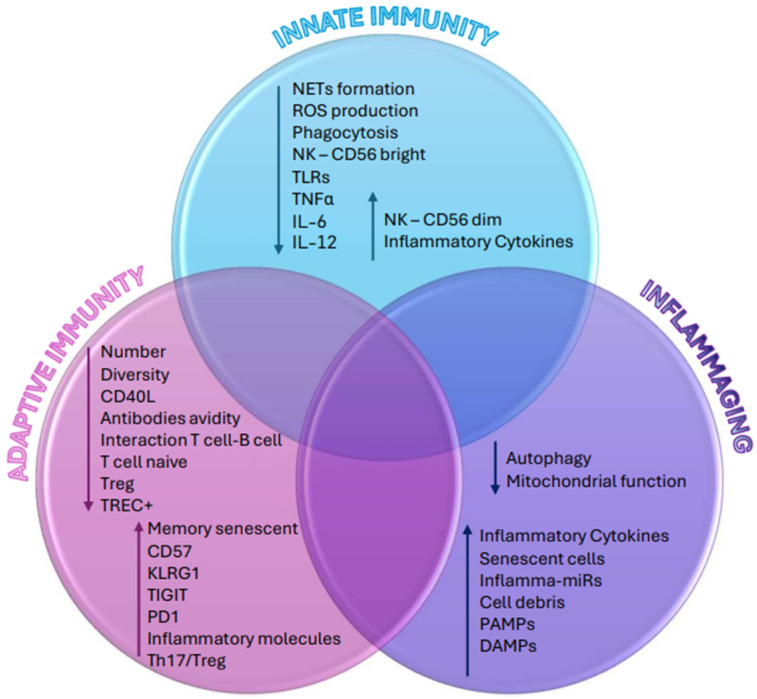
Immunological alterations observed during immunosenescence. ↑: increased level/activity; ↓: decreased level/activity.

Apart from immunosenescence, other fundamental molecular pathways that lead to inflammaging have been documented:

- Senescent cells accumulated within various tissues release pro-inflammatory mediators that have the potential to spread the senescent phenotype to neighboring cells, thereby contributing to age-related inflammation;
- There is an increase in cell debris levels from cell death or damage. Examples of these components include nucleic acids, mitochondrial DNA (mtDNA), cardiolipin, mitochondria, and heat-shock proteins. “Garb-aging” refers to the build-up of DAMPs with aging, which can lead to innate immunity and the release of pro-inflammatory cytokines;
- Age-related declines in autophagy and proteasome activity play a role in the accumulation of misfolded protein aggregates. Consequently, these aggregates serve as triggers for inflammatory pathways.
- The presence of pro-inflammatory circulating microRNA (inflammaMIR) and the age-related accumulation of agalactosylated N-glycans in the blood (one of the most potent indicators of human biological age);
- Nuclear DNA damage and telomere shortening caused by ROS and other agents set off a DNA repair response and stimulate the synthesis of pro-inflammatory molecules;
- Impaired regulation of the complement pathway can cause a local inflammatory response;
- The excessive availability of energy and nutrients can fuel an inflammatory process mediated by metabolic cells, a phenomenon known as “metaflammation”;
- The dysbiosis of the gut microbiota associated with aging represents a prominent source of inflammatory triggers [54,59,60,61,62,63,64,65,66,67,68] (Figure 1).

## 4. Immunosenescence in Multiple Sclerosis

### 4.1. Immunosenescence in Experimental Autoimmune Encephalomyelitis Models

Experimental autoimmune encephalomyelitis (EAE) is an animal model used for research in multiple sclerosis that offers significant insights into immunopathological mechanisms. However, notable differences exist between the immunopathology of EAE and MS: in EAE, CD4+ T cells are dominant, while in MS lesions, CD8+ T cells and macrophages are more common. Central tolerance, governed by the autoimmune regulator Aire in medullary thymic epithelial cells (TECs), induces the ectopic expression of self-antigens, allowing some autoreactive T cells to escape central tolerance and to be controlled by peripheral tolerance mechanisms, including Treg cells. Age-related interactions between Aire and Treg cells influence EAE susceptibility, indicating that peripheral tolerance may decline with age. The literature suggests that young Aire-knockout (KO) mice are resistant to EAE due to higher peripheral Treg cell levels, whereas older Aire-KO mice experience severe disease without changes in peripheral Treg cell numbers. Moreover, the age-related elevation of molecules such as nicotinamide adenine dinucleotide phosphate (NADPH) oxidase, matrix metalloproteinases (MMPs), and cell adhesion molecules suggests their involvement in BBB disruption, autoreactive T cell infiltration, disease progression, and neurodegeneration. Age also affects regeneration, with older animals showing reduced remyelination efficiency compared with younger ones. Age-related neurological deficits in MS (limb weakness and paralysis) have been modeled in animals through inoculation. The induction of EAE is species- and strain-specific and highly dependent on the age at inoculation, emphasizing that age-related factors significantly impact EAE pathogenesis [11,69,70,71].

### 4.2. Particularities of Immunosenescence in Multiple Sclerosis

In MS patients, inflammatory processes associated with immunosenescence can alter the function of CNS resident cells, inducing senescence and a pro-inflammatory state. The senescence-associated secretory phenotype (SASP) is a pivotal aspect of cellular senescence, which is particularly relevant in the context of multiple sclerosis (Table 4). In MS lesions, senescent cells exhibit a stable arrest in their cell cycle and actively secrete pro-inflammatory cytokines, chemokines, growth factors, and reactive oxygen species. The SASP actively contributes to MS pathology by influencing the behavior of neighboring cells, notably oligodendrocyte precursor cells, in a paracrine manner. Components of the SASP released by senescent cells, such as TNF-α, IL-1β, FGF2, and TGF-β, have various roles in MS. TNF-α and IL-1β, for instance, are known to promote OPC proliferation and differentiation but can also contribute to neuroinflammation when chronically elevated. Conversely, FGF2, essential for normal oligodendrocyte development and remyelination, may have dysregulated effects when released by senescent cells. TGF-β plays dual roles by both regulating myelin development and potentially inducing cell cycle arrest in surrounding cells, which could impair OPC function and hinder remyelination. The presence of senescent glial cells and neurons in MS lesions underscores their role as alternative sources of inflammation independently of traditional immune responses [54,72,73,74].

Age-related reductions in bone marrow cellularity and mesenchymal stromal cell proliferative ability, crucial for hematopoiesis, are notably pronounced in primary progressive and secondary progressive MS (PPMS and SPMS). While CD34+ HSC counts remain steady, the frequency of colony-forming cells is low in MS patients. Also, thymic involution is accelerated in these patients, as indicated by consistently lower TREC levels compared with age-matched healthy controls [13,75,76,77,78,79].

Regarding the adaptive immune system, it has been described that the CD8 T-cell compartment of young MS patients exhibits early immunological aging, with alterations in immunoregulatory and co-stimulatory molecules resembling those seen in elderly healthy individuals. Specifically, young patients with PPMS show a significant decline in the coinhibitory markers KLRG1 and LAG3 and an increase in the co-stimulatory molecule CD226, suggesting that progressive MS patients age more rapidly than those with relapsing–remitting MS (RRMS) [78,79,80,81,82,83,84,85].

Moreover, B cells play a critical role in MS progression by producing pro-inflammatory cytokines (TNF, lymphotoxin alpha, IL-6, granulocyte–macrophage colony-stimulating factor) and chemokines via the NF-kB pathway. In healthy individuals, cerebrospinal fluid (CSF) levels of TNF-α, CXCL10, and IL-8 increase with age, while IL-10 levels are low in the middle-aged population. In MS, premature immunosenescence may cause a decrease in IL-10 production 10–20 years earlier. IL-6 and TNF-α levels rise in both blood and CSF in RRMS and SP/PPMS, with serum TNF-α correlating with disease progression in PPMS, and IL-6 levels correlating with the disease duration [54,80].

Regarding innate immunity, patients with the RRMS phenotype exhibit circulating neutrophils resistant to apoptosis, increased inflammatory markers, and NETosis. New inflammatory lesions are linked to elevated levels of chemokines and enzymes, such as CXCL1, CXCL8, and elastase. During the early stages of relapses, neutrophils are found in the CSF, although their numbers tend to decrease as the disease progresses [54,79,80].

As we mentioned before, physiological aging is associated with an increase in CD56^dim^ NK cells and a decrease in CD56^bright^ NK cells. In the context of multiple sclerosis, studies have suggested that CD56^bright^ NK cells have beneficial effects, particularly in patients undergoing immunomodulatory treatment. For example, the drug daclizumab has been shown to expand the population of CD56^bright^ cells, which correlates with a reduction in gadolinium-enhancing lesions [11,38,43,48,49].

Senescent microglia secrete higher amounts of TNF-α and IL-6, and they have reduced migration and phagocytic abilities. This dysfunction leads to energy failure, decreased myelin debris clearance, impaired remyelination, the loss of neurotrophic support, and the release of neurotoxic substances, ultimately causing irreversible neurodegeneration. Activated microglia significantly contribute to the progressive phase of the disease by promoting oxidative stress [78,81].

Immune-reactive OPCs express genes for MMP9, the immunoproteasome, and IL1b, contributing to early blood–brain barrier (BBB) disruption and increased neuroinflammation, which leads to functional impairment. Progressive MS is marked by compartmentalized CNS inflammation with an intact BBB, while relapsing–remitting MS features a disrupted BBB, allowing peripheral immune cell invasion into the CNS. The transition from relapsing–remitting MS to progressive MS is marked by a shift from active inflammatory lesions driven by peripheral lymphocytes to chronic lesions with activated microglia. Therefore, we can affirm that in younger RRMS patients, an inflammatory attack on white matter can originate from the periphery due to focal cerebrovascular permeability involving interactions between CNS resident cells and hematogenous leukocytes. In contrast, we can conclude that in older progressive MS patients, most of the inflammatory cells cause remote damage through soluble substances that diffuse into surrounding tissues, subsequently activating microglia and astrocytes [54,80,86,87,88,89,90,91].

MS patients experience the remyelination of axons that survive inflammatory demyelination. This process has a neuroprotective effect, preventing the axonal degeneration associated with chronic demyelination. It also restores impulse transmission along axons in demyelinating plaques. The shift from the recurrent to the progressive MS phenotype correlates with age-related declines in remyelination. Remyelination is more common in the early stages of the disease but diminishes over time. Studies show that remyelination occurs in 40% of acute MS plaques but is absent in 89% of smoldering plaques. This explains the histologic and radiographic characteristics of MS plaques that change with disease duration. After approximately 30 years of disease progression, active plaques are nearly absent, with most plaques being dormant, smoldering, or largely remyelinated shadow plaques. Smoldering plaques typically start to appear around ten years into the disease and peak at around fifty years of age, usually coinciding with the clinical transition from relapse to progressive MS [54,75,76,77,78,79,80,81].

All these cellular and molecular mechanisms of the immune systems presented above lead, in the end, to neurodegeneration and significant brain atrophy. It is well established that all these factors effectively cause the MS brain to age prematurely. The rate of brain shrinkage in MS patients is about 0.7–1% per year, compared with 0.1–0.3% annually in healthy individuals [54,90,91].

## 5. Immunosenescence and Treatment Strategies

### 5.1. Current Treatments

As multiple sclerosis progresses with age, it becomes plausible that disease-modifying therapies (DMTs) may vary in their effectiveness due to evolving disease mechanisms. To gain comprehensive insights, randomized controlled trials encompassing all age groups and MS phenotypes would be ideal. However, the majority of Phase 3 clinical trials impose a maximum age limit of 55 at randomization, thus limiting our understanding of how DMTs perform in older populations [76].

Research indicates that the efficacy of DMTs diminishes with age (Table 5). Thus, for individuals with MS who are over 55 and have not experienced recent relapses, their therapeutic options include the following: maintaining the current DMT, de-escalating their therapy (transitioning to less potent DMTs as the disease activity decreases and risks increase or reducing doses or extending dosing intervals), or ceasing DMT use entirely [92,93,94,95,96,97,98].

Younger patients with recent relapses or active lesions are at a significantly higher risk of new relapses or imagistic activity de-escalation or discontinuation of therapy is considered. A recent study developed a 6-point validated scoring system to assess the risk of disease activity recurrence, assigning points for items such as age, number of lesions at discontinuation, and years without relapse. The risk of disease reactivation over five years was approximately 10% for those scoring up to 1 point, 40% for those with scores of 2 and 3 points, and 90% for those scoring 4 and 5 points (Table 6) [92,93].

The relationship between disability progression and DMT discontinuation remains unclear. The data in the literature on this topic are limited and contradictory. In a recent trial analyzing patients aged 55 and older, it was not conclusively determined that discontinuing treatment is non-inferior to continuation, but new relapses and radiographic lesions were rarely noticed in these studies. Similar findings were observed in MS patients discontinuing injectable DMTs after a long relapse-free period in another recent trial. These patients had comparable relapse rates to those continuing treatment but faced a higher risk of disability progression. A significant concern is the limited data on discontinuing highly effective therapies, particularly those that block immune cell trafficking, such as fingolimod and natalizumab. Discontinuation of these therapies can lead to a rebound effect with increased disease activity. Therefore, de-escalation, rather than complete cessation, is suggested for patients at higher risk of disease activity recurrence [76,92,93,94,95,96,97].

There are several potential benefits to discontinuing DMTs in suitable individuals; in particular, the reduction of adverse effects associated with these therapies (Table 7). Administering immunosuppressive DMTs to elderly patients with MS raises significant concerns due to the heightened risk of infections, particularly as the disability progresses. These medications alter the immune cell distribution and function (e.g., ocrelizumab is known to reduce levels of IgM and IgG antibodies). A critical complication is JC polyomavirus-associated progressive multifocal leukoencephalopathy (PML), which is especially concerning in older patients treated with natalizumab, but which has also been reported with dimethyl fumarate and fingolimod. Also, it is thought that aging and DMT use are associated with increased risks of reactivating HSV1 and VZV infections, possibly due to immunosenescence [76,92,99,100].

Older age is a significant risk factor for cancer development, and immunosenescent changes are thought to contribute to tumorigenesis (Table 7). These changes, including decreased T cell CD28 expression, increased program death ligand 1 expression by DCs, and reduced pro-inflammatory cytokines, create an immunosuppressive environment favorable for cancer growth. Evidence from various cohorts suggests a higher overall incidence of malignancies such as colorectal, prostate, and breast cancers in MS patients compared with the general population. The initiation of DMTs may further modulate immune responses and potentially increase the cancer risk. While interferons and glatiramer acetate have not shown increased cancer risk in clinical trials, other DMTs like natalizumab, fingolimod, and ocrelizumab have been associated with higher cancer incidences [76,101,102,103,104].

There are currently no established guidelines for when to recommend discontinuing or de-escalating DMTs. Some consider these options for individuals with a relapsing phenotype who are older than 50 and who have experienced at least 5 years of stability, or for patients with disability progression over several years, regardless of relapse history and despite ongoing treatment. We consider that the moment of de-escalation or discontinuation of DMTs should involve an approach based on a shared decision with the patient. Many patients tend to be reluctant to stop a therapy, so it is crucial to reassure them that this step represents a change in the MS therapeutic management, not a discontinuation of care, as this could lead to patient disengagement from the medical team. It is important to encourage ongoing interaction with the healthcare team and periodical clinical examinations and MRI imaging, focusing on their quality of life, exercise, symptom control, treatment of comorbidities, and hope for future advancements through ongoing research [92,93,94,95,96,97].

### 5.2. Future Prospectives

A potential strategy against cellular senescence involves two main approaches: senolysis, which targets the selective elimination of senescent cells to reduce their effects on tissues, and senomorphism, which aims to block the expression of the SASP and other harmful mediators associated with senescence. These approaches are intended to reduce the adverse effects of senescent cells, thereby preventing the associated chronic inflammation as well as the loss of neuroaxonal function and structure. Various agents are actively being researched to specifically eliminate senescent cells. Despite releasing pro-apoptotic factors as part of their SASP, senescent cells are resistant to apoptosis. Senolytics target the pro-survival pathways of senescent cells, known as senescent cell anti-apoptotic pathways (SCAPs). Cellular senescence and mitochondrial dysfunction, both hallmarks of aging, are closely intertwined; the upregulation of SCAP is associated with senescence-associated mitochondrial dysfunction (SAMD), which, in turn, perpetuates cellular senescence. Currently, six distinct SCAPs have been identified: the Bcl-2/Bcl-XL family, PI3K/Akt/ROS protective/metabolic pathways, p53/p21/serpine pathways, ephrins/dependence receptors/tyrosine kinases, HIF-1α, and heat shock protein 90 (HSP-90). Several senolytic drugs are being developed based on disrupting these SCAP mechanisms (Table 8) [11,105,106].

The combination of the tyrosine kinase inhibitor dasatinib and quercetin has been shown to reduce the accumulation of senescent cells and to improve survival in older mice. Additionally, these drugs have demonstrated immunomodulatory effects in MS when administered individually. Recent studies have highlighted that the periodic oral administration of dasatinib and quercetin as a senolytic cocktail can decrease naturally occurring senescent cell numbers, alleviate physical impairments, and extend the lifespan in aged mice. The effectiveness of these molecules as senolytics is further demonstrated by findings from a trial that investigated their role in eliminating senescent neurons in models of tau-dependent neurodegeneration. For example, in the context of Alzheimer’s disease (AD), targeting senescent OPCs associated with Aβ plaques in the APP/PS1 mouse model led to reduced neuroinflammation and improved cognitive function. The study revealed that these interventions not only reduced the presence of senescent neurons but also prevented brain atrophy, highlighting their potential therapeutic benefit in neurodegenerative diseases [11,105,106,107,108].

Navitoclax, also known as ABT263, is a significant senolytic drug capable of inducing apoptosis in senescent bone marrow hematopoietic stem cells. Its use has been associated with a reduction in myeloid bias and an improvement in hematopoietic function. In the context of neurodegenerative diseases, navitoclax has shown promise in various models. Navitoclax has also been effective in preventing tau-dependent pathology in models of tau-related neurodegeneration, ultimately leading to improved cognitive performance in experimental studies [105,106,107,108].

Treatment with rapamycin has been shown to alter the senescent phenotype of neural progenitor cells (NPCs) derived from induced pluripotent stem cells of progressive MS patients. It has been proved that rapamycin enhances NPCs’ ability to facilitate oligodendrocyte precursor cell differentiation in vitro. This indicates that senomorphic therapies have the potential to promote remyelination. Rapamycin treatment also restored astrocytes’ capacity to support OPC differentiation, suggesting that astrocytic senescence may also hinder myelin repair processes. These findings underscore the reversibility of cellular senescence with rapamycin treatment, highlighting its therapeutic potential in MS and other neurodegenerative conditions [11,83,107,108,109].

An intermittent fasting diet (which is proven to reduce autoreactive T cells and promote Treg cell production) and metformin treatment have been shown to effectively reverse the senescent state observed in rat OPCs. Metformin has demonstrated the ability to improve the differentiation and maturation processes of OPCs in aged rodents, thereby enhancing their capacity for remyelination. These interventions collectively contribute to the restoration of remyelination and neuroregeneration in animal models, underscoring their potential therapeutic benefits for conditions associated with impaired myelin repair [11,77,83,110,111].

Recent advancements aim to address thymic aging and its impact on immune function. Strategies targeting the FOXN1 transcription factor, which is crucial for thymic epithelial cell differentiation and tolerance induction, show promise in reversing thymic involution. This approach could potentially restore thymic integrity, thereby enhancing thymopoiesis and mitigating the thymic dysfunction observed in MS. It is known that an age-related decline in IL-7 expression contributes to thymic involution, affecting thymocyte maturation and TCR diversity. IL-7 supplementation has been shown to promote thymic recovery and increase naive T cell numbers. Of note, IL-22 enhances thymic recovery in autoimmune contexts and the keratinocyte growth factor supports TEC maintenance, both demonstrating efficacy in restoring thymopoiesis in aged mice [11,112,113].

Simvastatin has demonstrated effectiveness in slowing brain atrophy and the progression of disability in multiple sclerosis trials. This neuroprotective impact is thought to occur through its senomorphic function: reducing the activation of p38MAPK and levels of TNF-alpha and GM-CSF. The positive outcomes observed with simvastatin in secondary progressive MS and optic neuritis suggest that statins may play a pivotal therapeutic role, particularly during phases of active inflammation of RRMS. Future studies are required to clarify whether these benefits are specific to simvastatin or if they extend to other statins as well [77,78,114].

Ibudilast is a selective inhibitor of phosphodiesterases, including PDE-3, PDE-4, PDE-10, and PDE-11. It is also a macrophage migration inhibitory factor that alters cyclic adenosine monophosphate signaling, which is crucial for regulating activated macrophages during inflammation. This suggests that ibudilast may influence the activities of microglia and macrophages. Additionally, its ability to penetrate the BBB indicates potential effects on localized inflammation in the CNS. Research has shown ibudilast’s ability to mitigate the decline in the magnetization transfer ratio (MTR) in slow enlarging lesions (SELs). While SELs themselves do not predict disability progression, their volumes correlate significantly with the clinical outcome. Given its substantial impact on SELs, which suggests effectiveness in reducing chronic active lesions and modulating inflammation while also mitigating brain atrophy, ibudilast has emerged as a promising candidate for Phase 3 trials in progressive MS [77,115].

## 6. Discussion

Immunosenescence significantly influences the pathogenesis and clinical course of multiple sclerosis. In MS, immunosenescence exacerbates chronic neuroinflammation by promoting the accumulation of senescent immune cells within the CNS; these cells exhibit altered phenotypes and impaired function, leading to increased oxidative stress, DNA damage, and mitochondrial dysfunction. We have noted that these cellular dysregulations not only exacerbate local inflammation but also impede critical repair mechanisms such as remyelination. Age-related changes in histone modifications and immune cell activation further contribute to the pro-inflammatory state observed in MS, influencing the disease trajectory and treatment responses. Premature immunosenescence markers are prevalent in MS patients, accelerating disease evolution and diminishing the therapeutic efficacy over a certain period of time. Neuroinflammation associated with immunosenescence in MS significantly contributes to neurodegenerative processes, resulting in progressive neuronal loss, thereby leading to brain atrophy and cognitive decline. Microglial senescence, alongside dysregulated cytokine production that intensifies oxidative stress and mitochondrial dysfunction, and the loss of remyelination ability mark the transition from relapsing–remitting to progressive MS phenotypes [10,11,12,13,54,76,77,83].

Managing MS in older adults presents unique challenges due to the dual considerations of DMTs and age-related immune decline. Consequently, tailoring treatment strategies in order to balance therapeutic benefits with potential risks becomes imperative in clinical practice. Although there are currently no established guidelines for DMT discontinuation or de-escalation, research suggests that the efficacy of DMTs diminishes with age. Therefore, individuals over 55 who have not experienced recent relapses may consider maintaining their current DMT, de-escalating the therapy, or discontinuing DMT administration altogether. Although data on the relationship between disability progression and DMT discontinuation are limited and contradictory, some studies indicate that discontinuing DMTs in older patients does not significantly increase the risk of new relapses or radiographic lesions, although it may pose a higher risk of disability progression. Concerns also exist regarding the discontinuation of highly effective therapies, which may pose a risk of rebound disease activity, also leading to accumulating disability. We are looking forward to ongoing trials in order to provide more data on DMT discontinuation outcomes, which will enhance our understanding and guide future clinical practices [11,54,76,77,83].

Emerging therapeutic approaches targeting immunosenescence, such as senolytic agents, hold promise in mitigating disease progression and supporting neuroprotection in aging MS populations. The enthusiasm for this new therapeutic approach is favored by the fact that some of these molecules are already approved for other medical purposes. For example, metformin has shown efficacy in enhancing remyelination, while simvastatin has demonstrated potential in slowing brain atrophy and disability progression in MS, and ibudilast has shown promise in reducing chronic active lesions and brain atrophy. Despite significant strides, challenges remain in understanding the complex mechanisms of immunosenescence in MS and translating these insights into clinical practice [11,54,76,77,83].

## 7. Conclusions

In conclusion, immunosenescence poses significant challenges to the management of multiple sclerosis. As advancements in medical care extend the lifespan of MS patients, the increasing prevalence of older individuals diagnosed with this disease necessitates a better understanding of how age-related immune alterations influence MS progression and treatment options. As previously mentioned, age-related shifts in immune cell populations, cytokine profiles, and compromised immune surveillance collectively contribute to a pro-inflammatory environment that exacerbates MS pathogenesis. By unraveling all these complexities, we hope to develop future therapeutic strategies that are tailored to mitigate the impact of immunosenescence on MS. We have found that longitudinal studies focusing on elderly MS cohorts are essential to achieving better outcomes. It is safe to say that advancing our understanding of immunosenescence is critical, especially with regard to autoimmune diseases, not only for developing new targeted therapies but also for personalizing existing management strategies in order to provide our patients with the most up-to-date high-standard care.

## Figures and Tables

**Table 1:** The impact of immunosenescence on adaptive immunity [11].

Adaptive Immunity Cell	Changes in Immunosenescence
T cell	Decreased number of naive T cells
	Increased memory T cell population
	Reduction in TCR repertoire diversity
	Diminished presence of the CD28 co-stimulatory molecule
	Increased expression of CD57, KLRG1, TIGIT, and PD1
	Distinct cytokine secretion profile (SASP)
	Shift toward cytotoxicity mediated by NK receptors
B cell	Decreased number of naive B cells
	Increased memory B cell population
	Reduction in BCR repertoire diversity
	Increase in the population of ABCs
	Increased autoimmune activity
	Increase in the population of age-associated B cells

T cell receptor (TCR); Tyrosine-based inhibitory motif domain (TIGIT); Senescence-associated secretory profile (SASP); B cell receptor (BCR); Age-associated B cell (ABC); Natural killer (NK).

**Table 2:** Classes of PPRs [34,35,36,37].

Type of Pattern-Recognition Receptors	Action
Toll-like receptors	NF-κB activation, activation of MAP kinases pathway to recruit pro-inflammatory cytokines and co-stimulatory molecules, which promote inflammatory responses
NOD-like receptors	Stimulation of the inflammasome complex and the production of IL-11, IL-18, and IL-33
Rig-like receptors	Proximal triggering of the IFN pathway

**Table 3:** Immunosenescence: innate immunity [11].

Innate Immunity Cell	Changes in Immunosenescence
Neutrophils	Reduced ROS production
	Impaired NET formation
	Altered adhesion and phagocytosis
Monocytes/Macrophages	Impaired migration and chemotaxis
	M2-like over classic M1-like macrophages
	Reduced TLR expression
NK Cells	Increased CD56^dim^ and decreased CD56^bright^ subset
	Reduced cytotoxic activity
	Defective degranulation capacity
Dendritic Cells	Increased ROS production
	Impaired phagocytic capabilities and antigen presentation
	Decreased responsiveness to TLR stimulation
	Reduced production of type I and II interferons
	Enhanced pro-inflammatory cytokine secretion

Toll-like receptor (TLR); Reactive oxygen species (ROS); Natural killer (NK); Neutrophil extracellular trap (NET).

**Table 4:** SASP molecules and their immune role [43].

SASP Molecule	Role
IL-1	Pro-inflammatory
IL-6	Pro-inflammatory activity contributing to inflammation and DNA damage
IL-7	Promotes B lymphocyte growth and T lymphocyte activation
IL-11	Hematopoiesis and tissue cell proliferation
IL-15	Necessary for T cell regulation; high levels are pro-inflammatory
IL-8	Chemotaxis and enhancement of pro-inflammatory activity
CXCL1	Neutrophil activation
MCP2	Monocyte chemotaxis
TNFα	Induces apoptosis
VEGF	Blood vessel formation
IGF-1	Proliferation and apoptosis

SASP: Senescence-associated secretory phenotype, IL: Interleukin, CXCL1: C-X-C motif chemokine ligand 1, MCP2: Monocyte chemotactic protein 2, TNFα: Tumor necrosis factor-alpha, VEGF: Vascular endothelial growth factor, IGF-1: Insulin-like growth factor 1.

**Table 5:** Impact of DMTs on the relapse rate and disability progression in different age groups [77,98].

Molecule	Cut-Off Age	Impact on Annual Relapse Rate Vs. Placebo	Impact on Disability Progression Vs. Placebo
Teriflunomide	<38 years	↓	↓
	≥38 years	↓	no impact
Dimethyl fumarate	<40 years	↓	↓
	≥40 years	↓	no impact
Fingolimod	<40 years	↓	no impact
	≥40 years	no impact	no impact
Siponimod	<50 years ≥50 years	n/a	↓ ↓
Cladribine	<40 years ≥40 years	↓ ↓	↓ ↓
Ocrelizumab	<45 years ≥45 years	↓ ↓	↓ ↓

↓: the molecule diminishes the Annual Relapse Rate/slows down Disability Progression.

**Table 6:** Vienna DMT discontinuation score [92,93].

	Value	Score
Age	<45 years	2 points
	45–55 years	1 point
	>55 years	0 points
MRI activity	≥3 new or enlarged T2 lesions or 1 gadolinium-enhancing lesion	2 points
	<3 new or enlarged T2 lesions and no gadolinium-enhancing lesion	1 point
Stable disease	<4 years	2 points
	4–8 years	1 point
	>8 years	0 points

**Table 7:** Increased risks of DMTs with age [77,99].

Treatment	Adverse Events Increased with Age
Fingolimod	Reduction of heart rate Hypertension HSV1/VVZ reactivation PML Malignancies (frequently affecting the skin)
Natalizumab	PML HSV1/VVZ reactivation
Cladribine	HSV1/VVZ reactivation Malignancies (frequently solid tumors)
Ocrelizumab	HSV1/VVZ reactivation Hypogammaglobulinemia Malignancies (frequently breast cancer) PML
Siponimod	Hypertension Diabetes Macular edema
Dimethyl fumarate	Lymphopenia PML (frequently related to grade 3 lymphopenia)

**Table 8:** SCAP mechanisms and senolytic drugs [106].

SCAP	Senolytic Drug
Bcl-2/Bcl-XL	Navitoclax A1331852 A1155463 Fisetin
PI3K/Akt/ROS	Quercetin Piperlongumine Fisetin
p53/p21/serpine	Quercetin Fisetin FOXO related molecule
Ephrins/dependence receptors/tyrosine kinases	Dasatinib Piperlongumine
HIF-1α	Quercetin Fisetin
HSP-90	Tanespimycin Geldanamycin Alvespimycin
